# Establishment and application of a TaqMan-based multiplex real-time PCR for simultaneous detection of three porcine diarrhea viruses

**DOI:** 10.3389/fmicb.2024.1380849

**Published:** 2024-04-16

**Authors:** Jing Ren, Congcong Zu, Yang Li, Meng Li, Jinyuan Gu, Fengling Chen, Xiaowen Li

**Affiliations:** ^1^Shandong Engineering Research Center of Swine Health Data and Intelligent Monitoring, Dezhou University, Dezhou, China; ^2^Shandong Key Laboratory of Biophysics, Institute of Biophysics, Dezhou University, Dezhou, China; ^3^Shandong New Hope Liuhe Agriculture and Animal Husbandry Technology Co., Ltd. (NHLH Academy of Swine Research), Dezhou, China

**Keywords:** multiplex real-time PCR, PEDV, PoRV, PDCoV, porcine viral diarrhea

## Abstract

**Introduction:**

Porcine viral diarrhea is a common clinical disease, which results in high mortality and economic losses in the pig industry. Porcine epidemic diarrhea virus (PEDV), porcine rotavirus (PoRV), and porcine deltacoronavirus (PDCoV) are important diarrhea viruses in pig herds. The similarities of their clinical symptoms and pathological changes make it difficult to distinguish these three viruses clinically. Therefore, there is a need for a highly sensitive and specific method to simultaneously detect and differentiate these viruses.

**Methods:**

A multiplex real-time PCR assay using TaqMan probes was developed to simultaneously detect PEDV, PoRV, and PDCoV. To assess the efficacy of the established assay, 30 clinical samples with diarrhea symptoms were used to compare the results obtained from the multiplex real-time PCR assay with those obtained from commercial singleplex real-time PCR kit. Importantly, a total of 4,800 diarrhea samples were tested and analyzed to validate the utility of the assay.

**Results:**

This multiplex real-time PCR assay showed high sensitivity, specificity, and excellent repeatability with a detection limit of 1 × 10^2^ copies/μL. Comparing the results of the commercial singleplex real-time PCR kit and the multiplex real-time PCR method for detecting PEDV, PoRV, and PDCoV, there was complete agreement between the two approaches. Clinical data revealed single infection rates of 6.56% for PEDV, 21.69% for PoRV, and 6.65% for PDCoV. The co-infection rates were 11.83% for PEDV + PoRV, 0.29% for PEDV + PDCoV, 5.71% for PoRV + PDCoV, and 1.29% for PEDV + PDCoV + PoRV, respectively.

**Discussion:**

The multiplex real-time PCR method established in this study is a valuable diagnostic tool for simultaneously differentiating PEDV, PoRV, and PDCoV. This method is expected to significantly contribute to prevent and control the spread of infectious diseases, as well as aid in conducting epidemiological investigations.

## 1 Introduction

Viral diarrheal disease poses a serious threat to the swine industry, causing substantial global economic losses (Wu et al., 2022; Hou et al., 2023). Numerous diarrhea viruses have been identified in swine, including transmissible gastroenteritis virus (TGEV), porcine epidemic diarrhea virus (PEDV), porcine delta coronavirus (PDCoV), porcine enteric alpha coronavirus (PEAV), porcine rotavirus (PoRV), porcine sapvirus (PSaV), porcine norovirus (PNoV), porcine teschenvirus (PTV), porcine kobuvirus (PKV), seneca valley virus (SVV), porcine reovirus (ReoV), porcine bocavirus (PBoV), porcine astrovirus (PAstV), and so on (Huang et al., 2019). Among these swine diarrhea viruses, PEDV, PoRV, and PDCoV are the most destructive pathogens causing anorexia, vomiting, diarrhea, and dehydration (Hou et al., 2023). With the rapid development of intensive aquaculture, co-infection or secondary infection with these viruses is prevalent, resulting in significant morbidity and mortality (Huang et al., 2019; Hou et al., 2023).

PEDV is an enveloped, single-stranded RNA virus that belongs to the *Alphacoronavirus* genus in the *Coronaviridae* family (Huang et al., 2013; Hu et al., 2021). The spherical PEDV particles vary in size, with an average diameter of ~130 nm (Hu et al., 2021). The PEDV genome is ~28 kb (Huang et al., 2013; Hu et al., 2021). While PEDV can infect pigs of any age, it is especially severe in piglets, often resulting in 100% morbidity and mortality (Shibata et al., 2000; Huang et al., 2013). The first reported cases of porcine epidemic diarrhea (PED) occurred in England in 1971, followed by a subsequent outbreak in Belgium in 1977 (Si et al., 2020; Hou et al., 2023). It was later identified in China in the 1980's (Wang et al., 2016). In December 2010, a new strain of PEDV with high virulence emerged in China, resulting in the death of over 1 million piglets and a 100% mortality rate among suckling piglets (Wang et al., 2016; Wu et al., 2022). Since then, the emerging and highly virulent strains of PEDV have gradually spread throughout the global swine industry, causing significant economic losses and becoming a leading cause of diarrhea in pigs (Wang et al., 2016; Hou et al., 2023).

PDCoV is a new swine enteric coronavirus that belongs to the newly identified genus *Deltacoronavirus* in the *Coronaviridae* family (Ma et al., 2015). It is an enveloped, single-stranded positive-stranded RNA virus with a genome size of ~25.4 kb. PDCoV was first detected in Hong Kong, China in 2012, and then in the United States in 2014 (Woo et al., 2012; Wang et al., 2014). The first outbreak of PDCoV occurred in the United States in 2014 and quickly spread to at least 20 states, resulting in significant economic losses in the global pig industry (Ma et al., 2015; He et al., 2020). PoRV is a non-enveloped, double-stranded RNA virus from the genus *Rotavirus* in *Reoviridae* family. Its genome is ~18,500 bp long, consisting of 11 segments of dsRNA (Chepngeno et al., 2020). PoRV is a major cause of diarrhea in piglets, particularly those aged 1–8 weeks (Chepngeno et al., 2020; Tao et al., 2023). It can also spread between pigs and humans, posing a significant threat to public health and safety in China (Li et al., 2018).

Pigs infected with PEDV, PoRV, and PDCoV exhibit similar clinical symptoms and pathological changes, making it hard to differentiate them. Moreover, the prevalence of co-infection and secondary infection among PEDV, PoRV, and PDCoV poses significant challenges in clinical diagnostics (Shu et al., 2022; Hou et al., 2023). It is therefore urgent to develop an efficient molecular method for distinguishing these viruses in the clinic. Real-time PCR utilizes fluorescence signals to monitor the PCR process in real-time, offering advantages in terms of speed, sensitivity, and reproducibility when compared to conventional PCR method. Hence, it is commonly used in clinical detection. However, singleplex real-time PCR is not convenient for the simultaneous detection of co-infection of multiple pathogens, and repeated detection of various pathogens wastes time and makes operations cumbersome (Ben Salem et al., 2010; Liu et al., 2019). Multiplex real-time PCR is a method that can detect multiple pathogens in a single reaction system (Huang et al., 2019; Li et al., 2022). In this study, we developed a multiple real-time PCR based on TaqMan probe to simultaneously and accurately detect PEDV, PoRV, and PDCoV. This assay exhibited high sensitivity and specificity for the target genes. Additionally, we used this method to detect and analyze 4,800 clinical samples collected from pig farms in multiple provinces of China, providing valuable data to help formulate prevention and control strategies in this particular region.

## 2 Materials and methods

### 2.1 Viruses, primers, and probes

The nucleic acid of various viruses and bacteria, including respiratory syndrome virus (PRRSV), pseudorabies virus (PrV), porcine circovirus (PCV1), PCV2, classical swine fever virus (CSFV), African swine fever virus (ASFV), *Streptococcus suis* (SS), *Salmonella enteritidis* (SE), PEDV, PoRV, and PDCoV were stored at −80°C in our laboratory until needed. At least 30 genome sequences of PEDV, PoRV, and PDCoV were downloaded from NCBI for analysis. After comparison, the M gene of PEDV and PDCoV, and the NSP3 gene of PoRV were found to be the most conserved gene sequences. Primers and probes were designed using Primer Premier 5 software (Premier, Canada) based on the most conserved region. TaqMan probes for PEDV, PoRV, and PDCoV were labeled with FAM, VIC, and Cy5 at the 5′ end, respectively, with all quenchers at the 3′ end being BHQ. The sequences of the primers and probes designed in this study are presented in Table 1. Primers and probes were synthesized by Sangon Biotech (Shanghai) Co., Ltd.

**Table 1:** Primers and probes designed for the multiplex real-time PCR.

**Virus**	**Primer/probe**	**Sequence (5^′^-3^′^)**	**Size (bp)**	**Target gene**
PEDV	Forward	CATCTGATTCTGGACAGTTG	226	M
	Reverse	CTATACACCAACACAGGCTC		
	Probe	(FAM)TTTCAGAGCAGGCTGCATAT(BHQ1)		
PoRV	Forward	ACCATCTACACATGACCCTCTATGAG	83	NSP3
	Reverse	ACATAACGCCCCTATAGCCATTTAG		
	Probe	(VIC)ACAATAGTTAAAAGCTAACACTG(BHQ1)		
PDCoV	Forward	ATCGACCACATGGCTCCAA	72	M
	Reverse	CAGCTCTTGCCCATGTAGCTT		
	Probe	(Cy5)CACACCAGTCGTTAAGCATGGCAAGCT(BHQ3)		

### 2.2 Construction of plasmid standards

The target fragments of PEDV, PoRV, and PDCoV were amplified separately using the HiScript II One Step RT-PCR Kit (Dye Plus; Nanjing Vazyme Biotech Co., Ltd.). The PCR fragments were purified and cloned into the pMD18-T vector (Takara Biomedical Technology (Beijing) Co., Ltd.). The transformed clones were then introduced into the E.coli DH5α strain. Positive clones were cultured and plasmid extraction was performed using the TaKaRa MiniBEST Universal Genomic DNA Extraction Kit [Takara Biomedical Technology (Beijing) Co., Ltd.]. The constructed plasmid was confirmed by DNA sequencing and used as the standard positive control. The copy number of recombination plasmids was calculated using the following formula (Li et al., 2022):


$$\begin{array}{l} Plasmid\;copies\text{/}\mu L=\frac{\left(\text{6.02}\times \text{1}{\text{0}}^{\text{23}}\right)\times \left(\text{X ng/}\mu L\times \text{1}{\text{0}}^{\text{-9}}\right)}{plasmid\;length\left(bp\right)\times \text{660}} \end{array}$$

A 10-fold series dilution was conducted for each plasmid, ranging from 1.0 × 10^8^ to 1.0 × 10^1^ copies/μL. Singleplex real-time PCR was performed for each virus using the 10-fold diluted plasmids to generate standard curve. For multiplex standard curves, each plasmid was diluted to 3.0 × 10^9^ copies/μL and pooled in equal volume to make a concentration of 1.0 × 10^9^ copies/μL for each plasmid. The pooled plasmid was then diluted in a 10-fold series, ranging from 1.0 × 10^8^ to 1.0 × 10^3^ copies/μL, to establish multiplex standard curves.

### 2.3 Reaction conditions of the singleplex real-time PCR

All real-time PCR reaction systems were set to a volume of 20 μL. For singleplex real-time PCR of PDCoV, RV, and PEDV, the reaction system included 10 μL 2× PerfectStart Probe One-Step qPCR SuperMix, 0.4 μL TransScript Probe One-Step RT Enzyme Mix (Transgen, Beijing), 0.4 μL each of forward/reverse primer (10 μM), 0.4 μL TaqMan probe (10 μM), 5.5 μL RNA template, and 2.9 μL nuclease-free water.

Amplification was performed on a Bio-Rad CFX96™ Real-time System (Bio-Rad, Hercules, CA, USA) using the following program: 45°C for 5 min, 94°C for 30 s, 40 cycles of 94°C for 5 s, and 52°C for 30 s. Fluorescence signal was automatically collected at the end of each cycle. All qPCR results were analyzed using CFX Manager™ software.

### 2.4 Optimization of multiplex real-time PCR assay

The concentrations of primers and probes were optimized as previously described (Pan et al., 2020). After repeated tests, the optimal reaction conditions for multiplex real-time PCR were determined as follows: 10 μL 2× PerfectStart Probe One-Step qPCR SuperMix, 0.4 μL TransScript Probe One-Step RT Enzyme Mix (Transgen, Beijing), 0.4 μL each of forward/reverse primer and probe (10 μM) for PoRV and PDCoV, 0.2 μL each of forward/reverse primer and probe (10 μM) for PEDV, 5.5 μL RNA template, 1.1 μL nuclease-free water, with a total reaction volume of 20 μL. The same instrument and real-time PCR program were used as described above.

### 2.5 Sensitivity, specificity, and repeatability test of the multiplex real-time PCR assay

To determine the limit of detection (LOD) for the multiplex real-time PCR method, the aforementioned pooled standard plasmids were diluted in a 10-fold serial manner, ranging from 1.0 × 10^3^ to 1.0 × 10^0^ copies/μL in nuclease-free water. These diluted standard plasmids were then used as templates for multiplex real-time PCR amplification. The reliable LOD was the lowest concentration that achieved a 95% positive detection rate.

To avoid false positives caused by other viruses or bacteria that may be present in the samples, two RNA viruses (PRRSV and CSFV), three DNA viruses (PCV1, PCV2, and ASFV), and two bacterias (*S. suis* and *S. enteritidis*) were used for specificity test of multiplex real-time PCR assay. Nucleic acids were extracted using the VAMNE Virus DNA/RNA Extraction Kit (Nanjing Vazyme Biotech Co., Ltd.). For RNA viruses, the cDNA was generated with the TransScript Probe One-Step qRT-PCR SuperMix (Beijing Transgen Biotech Co., Ltd.). These DNA and cDNA samples were first detected using commercial kits, and positive samples with CT values <25 were selected as template for specificity test of the multiplex real-time PCR assay. The standard plasmids of PEDV, PoRV, and PDCoV were used as positive controls, while nuclease-free water served as the negative control.

To assess its repeatability of the multiplex real-time PCR, a 10-fold serially diluted standard template was used, ranging from 1.0 × 10^8^ to 1.0 × 10^3^ copies/μL. Each reaction was performed with three replicates. Intra-assay repeatability was determined by conducting three simultaneous detections of each plasmid under identical conditions. Inter-assay repeatability was evaluated by repeating the assays three times individually at different time points. The coefficient of variation (CV) of the Cq values from the three experiments was calculated to estimate the level of repeatability. Data analysis was conducted using Microsoft Excel.

### 2.6 Verification of the multiplex real-time PCR assay

Our multiplex real-time PCR method and a commercial singleplex real-time PCR Kit were used to simultaneously detect 30 clinical samples with diarrhea symptoms. The detection results obtained from our established methods were then compared with those obtained from singleplex real-time PCR.

### 2.7 Clinical application of the multiplex real-time PCR assay

A total of 4,800 samples including fecal samples (*n* = 2,341), rectal swabs (*n* = 1,874), oral fluid (*n* = 145), and oropharyngeal swabs (*n* = 440), were used to investigate the prevalence of PEDV, PoRV, and PDCoV using the multiplex real-time PCR assay. These samples were collected from pig farms located in numerous provinces of China, including Gansu, Shanxi, Shaanxi, Shandong, Henan, Hubei, Anhui, Zhejiang, Jiangsu, Guangdong, Guangxi, Guizhou, Sichuan, Hunan, Chongqing, and Jiangxi, during the period from November 2021 to July 2022.

Clinical samples were treated with phosphate buffer saline (PBS), and the supernatant was collected after vortexing and centrifugation. Nucleic acids were extracted using the VAMNE Virus DNA/RNA Extraction Kit (Nanjing Vazyme Biotech Co., Ltd.). Reverse transcription was done with the TransScript Probe One-Step qRT-PCR SuperMix (Beijing Transgen Biotech Co., Ltd.). The constructed plasmid and nuclease-free were employed as positive and negative controls, respectively. All samples were tested with the multiplex real-time PCR assay to determine virus positivity. Infection rates were analyzed after obtaining assay results for all clinical samples.

## 3 Results

### 3.1 Single real-time PCR assay for individual virus

**Figure 1:**
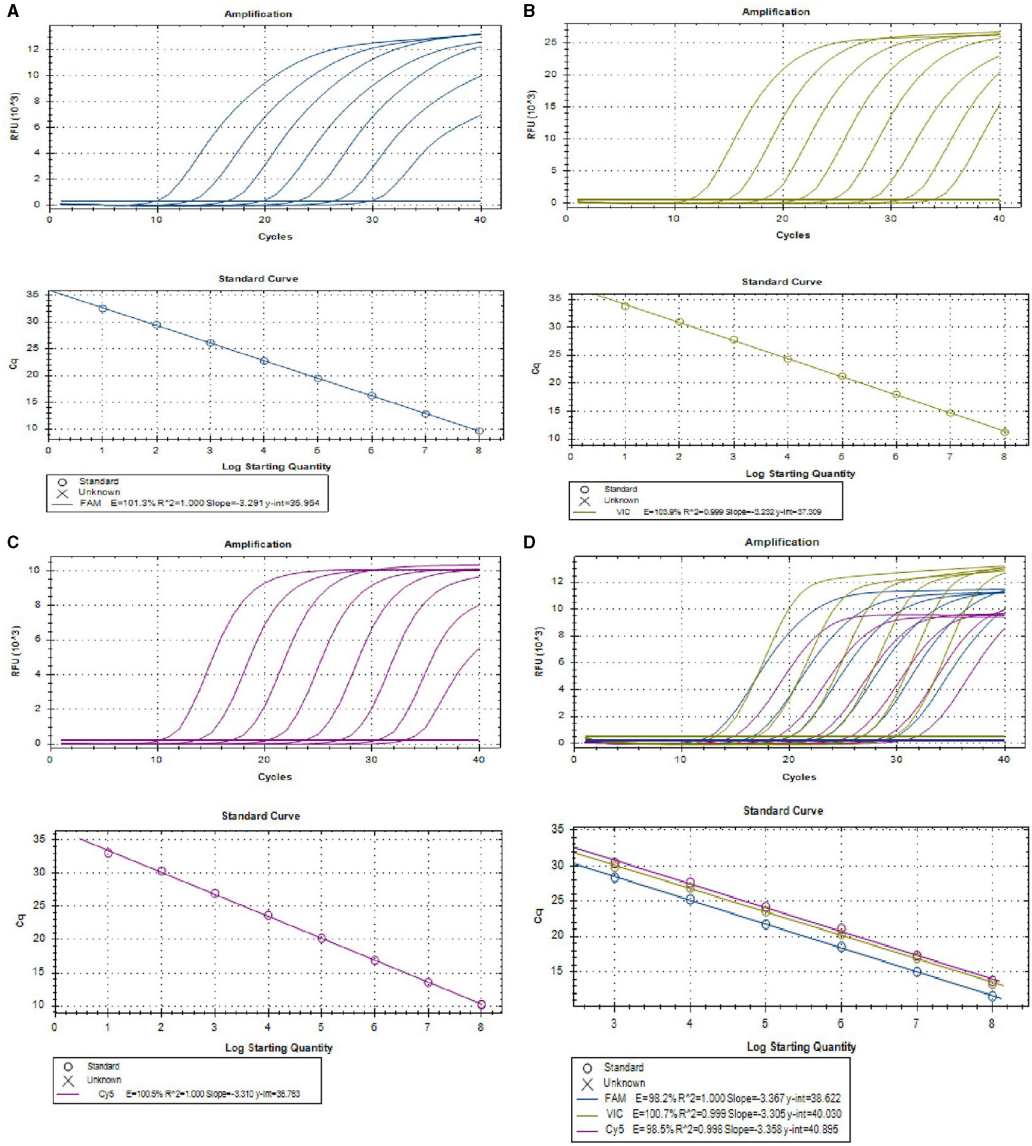
The amplification curves **(top)** and the standard curve **(bottom)** of the single and multiplex real-time PCR assay. **(A–C)** The amplification curves **(top)** and the standard curve **(bottom)** of single real-time PCR assay for detection of PEDV **(A)**, PoRV **(B)**, and PDCoV **(C)**, respectively, with concentrations of each plasmid standard ranging from 1.0 × 10^8^ to 1.0 × 10^1^ copies/μL. **(D)** Amplification curves **(top)** and standard curves **(bottom)** of optimized multiplex real-time PCR for simultaneous detection of PEDV, PoRV, and PDCoV. The concentrations of each plasmid standard are from 1.0 × 10^8^ to 1.0 × 10^3^ copies/μL.

To develop a multiplex real-time PCR, we first established singleplex real-time PCR for each virus using different fluorescence-labeled target probes. Standard curve for each virus was created using 10-fold serial dilutions of plasmids ranging from 1.0 × 10^8^ to 1.0 × 10^1^ copies/μL. All the standard curves showed excellent correlation coefficients and amplification efficacy, with PEDV (*R*^2^ = 1.000; E = 101.3%), PoRV (*R*^2^ = 0.999; E = 103.9%), PDCoV (*R*^2^ = 1.000; E = 100.5%), respectively. This indicates that our plasmid standards were qualified, and the designed primers and probes were efficient (Figures 1A–C).

### 3.2 Establishment of multiplex real-time PCR assay

Serial dilutions of mixed viral standard plasmids were tested with the using the optimized multiplex assay. The results demonstrated that the multiplex real-time PCR successfully detected all target genes of these three viruses (Figure 1D). The standard curves exhibited excellent correlation coefficients and amplification efficacy for each virus, for details, PEDV (*R*^2^ = 1.000; E = 98.2%), PoRV (*R*^2^ = 0.999; E = 100.7%), and PDCoV (*R*^2^ = 0.998; E = 98.5%; Figure 1D), indicating the validity and reliability of the multiplex real-time PCR.

### 3.3 The specificity of the multiplex real-time PCR assay

**Figure 2:**
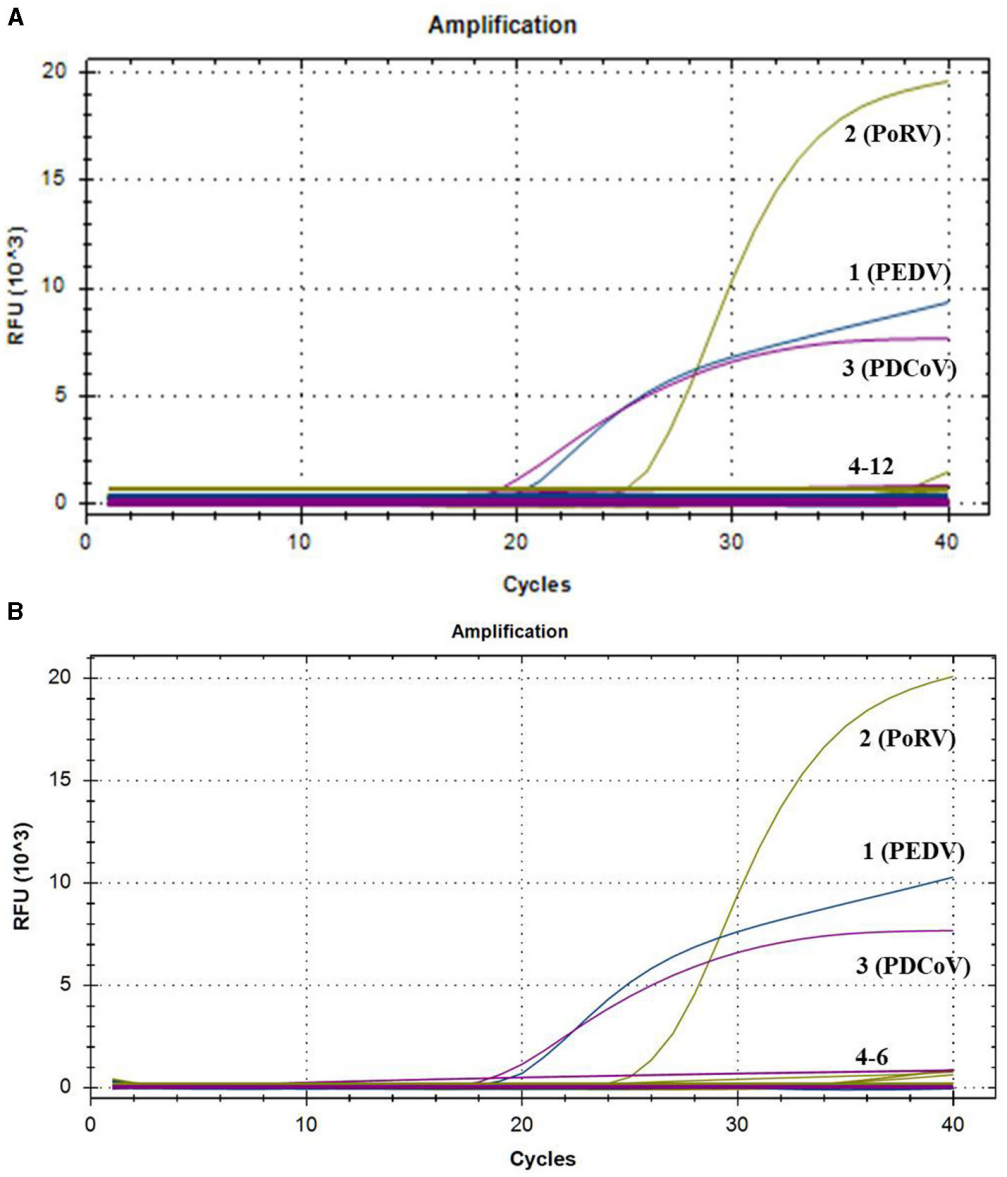
The amplification curves of specificity tests of multiplex real-time PCR. Only PEDV, PoRV, and PDCoV showed positive fluorescence signals, while other swine pathogens and clinical samples from healthy pigs exhibited no fluorescence signals. **(A)** 1–3: positive templates of PEDV, PoRV, and PDCoV. 4–12: negative control and positive templates of PRRSV, PRV, PCV1, PCV2, CSFV, ASFV, SS, and SE. **(B)** 1–3: positive templates of PEDV, PoRV, and PDCoV. 4–6: templates of clinical samples from healthy pigs.

To evaluate the specificity of the multiplex real-time PCR, the positive samples of PRRSV, PRV, PCV1, PCV2, CSFV, ASFV, SS, and SE were used as templates for amplification with this multiplex system. The standard plasmids of PEDV, PoRV, and PDCoV were tested as the positive controls, while nuclease-free water was tested as negative control. As shown Figure 2A, all the target pathogens were successfully detected, while no positive signal was observed from other eight viruses and negative control. Additionally, three clinical samples from healthy pigs were further tested to confirm the specificity. Amplification curves also demonstrated that only target genes from the standard plasmids were detected, with no positive signal detected in the clinical samples from healthy pigs (Figure 2B). These findings indicated that the multiplex real-time PCR assay was highly specific.

### 3.4 The sensitivity of the multiplex real-time PCR assay

To access the sensitivity of this multiplex real-time PCR assay, we tested pooled standard plasmids with concentrations ranging from 1.0 × 10^3^ to 1.0 × 10^0^ copies/μL under optimized reaction conditions. The method was able to detect positive samples with concentrations as low as 1.0 × 10^1^ copies/μL (Table 2). However, further experiments showed the detection rate for samples at 1.0 × 10^1^ copies/μL was < 95% of replicates (Table 2). Therefore, the reliable limit of detection for this method is 1 × 10^2^ copies/μL.

**Table 2:** The sensitivity tests of multiplex real-time PCR.

**Templates**	**Concentrations (copies/μL)**	**The number of tested samples**	**Positive number**	**Positive rate (%)**	**95% confidence region**
PEDV	1 × 10^3^	30	30	100	√
	1 × 10^2^	30	30	100	√
	1 × 10^1^	30	28	93.3	×
	1 × 10^0^	30	0	0	×
	Negative control	30	0	0	×
PoRV	1 × 10^3^	30	30	100	√
	1 × 10^2^	30	30	100	√
	1 × 10^1^	30	27	90.0	×
	1 × 10^0^	30	0	0	×
	Negative control	30	0	0	×
PDCoV	1 × 10^3^	30	30	100	√
	1 × 10^2^	30	30	100	√
	1 × 10^1^	30	28	93.3	×
	1 × 10^0^	30	0	0	×
	Negative control	30	0	0	×

### 3.5 Repeatability of the multiplex real-time PCR assay

The multiplex real-time PCR assay was confirmed to be repeatable by detecting standard plasmids at concentrations ranging from 1.0 × 10^8^ to 1.0 × 10^3^ copies/μL. As shown in Table 3, the variation coefficients (CVs) of Cq values in the intra-group and inter-group reproducibility tests were between 0.11 and 0.87% and between 0.11 and 1.63%, respectively. These results indicated that the multiplex real-time PCR assay established in this study had satisfactory repeatability.

**Table 3:** The repeatability tests of multiplex real-time PCR.

**Templates**	**Concentrations (copies/μL)**	**Intra-assay**		**Inter-assay**	
**Cq values (mean** ±**SD)**	**CV%**	**Cq values (mean** ±**SD)**	**CV%**
PEDV	1 × 10^8^	11.53 ± 0.05	0.40	11.41 ± 0.16	1.38
	1 × 10^7^	15.45 ± 0.09	0.55	15.30 ± 0.15	0.98
	1 × 10^6^	19.00 ± 0.13	0.68	18.84 ± 0.18	0.96
	1 × 10^5^	22.19 ± 0.05	0.21	21.93 ± 0.17	0.78
	1 × 10^4^	25.75 ± 0.11	0.43	25.31 ± 0.14	0.56
	1 × 10^3^	29.02 ± 0.15	0.50	28.61 ± 0.11	0.38
	Negative control	ND	ND	ND	ND
PoRV	1 × 10^8^	12.96 ± 0.11	0.85	12.89 ± 0.21	1.63
	1 × 10^7^	16.77 ± 0.15	0.87	16.93 ± 0.23	1.33
	1 × 10^6^	20.03 ± 0.06	0.29	20.16 ± 0.18	0.88
	1 × 10^5^	23.18 ± 0.03	0.15	23.23 ± 0.16	0.68
	1 × 10^4^	26.66 ± 0.08	0.11	26.59 ± 0.35	1.33
	1 × 10^3^	29.53 ± 0.11	0.39	29.58 ± 0.16	0.52
	Negative control	ND	ND	ND	ND
PDCoV	1 × 10^8^	13.36 ± 0.11	0.83	13.73 ± 0.02	0.11
	1 × 10^7^	17.39 ± 0.14	0.80	17.47 ± 0.04	0.22
	1 × 10^6^	21.03 ± 0.03	0.14	21.10 ± 0.03	0.14
	1 × 10^5^	24.15 ± 0.10	0.42	24.20 ± 0.06	0.25
	1 × 10^4^	27.72 ± 0.16	0.56	27.61 ± 0.06	0.23
	1 × 10^3^	30.37 ± 0.04	0.12	30.58 ± 0.23	0.76
	Negative control	ND	ND	ND	ND

ND means no data.

### 3.6 Verification of the multiplex real-time PCR assay by commercial singleplex real-time PCR kit

Thirty clinical samples were used to compare the results of the multiplex real-time PCR with commercial singleplex real-time PCR kit. As shown in Supplementary Table 1, the results of the multiplex real-time PCR were consistent with the commercial singleplex real-time PCR, suggesting that it can effectively replace the commercial singleplex real-time PCR for simultaneous detection of PEDV, PoRV, and PDCoV.

### 3.7 Clinical application of the multiplex real-time PCR

**Figure 3:**
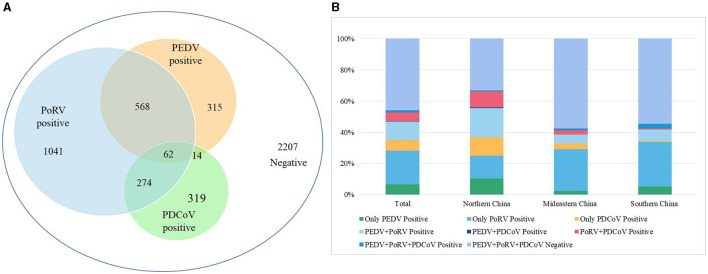
The detection results of clinical diarrhea samples using the multiplex real-time PCR assay established in this study. **(A)** The detection results of 4,800 clinical diarrhea samples. **(B)** A comparison of positive rates from different regions of China. These 4,800 clinical diarrhea samples were collected from 16 provinces of China, which were divided into three regions: Northern China (Gansu, Shanxi, and Shaanxi), Mideastern China (Shandong, Henan, Hubei, Anhui, Zhejiang, and Jiangsu), and Southern China (Guangdong, Guangxi, Guizhou, Sichuan, Hunan, Chongqing, and Jiangxi).

A total of 4,800 clinical samples were detected using the multiplex real-time PCR assay established in this study. As shown in Figure 3A, the single infection rates for PEDV, PoRV, and PDCoV were 6.56% (315/4,800), 21.69% (1,041/4,800), and 6.65% (319/4,800), respectively. The co-infection rates for PEDV + PoRV, PEDV + PDCoV, and PoRV + PDCoV were 11.83% (568/4,800), 0.29% (14/4,800), and 5.71% (274/4,800), respectively. The mixed infection rate for PEDV + PDCoV + PoRV was 1.29% (62/4,800).

These 4,800 clinical samples were collected from 16 provinces which were divided into three regions based on the geographical distribution: Northern China (Gansu, Shanxi, and Shaanxi), Mideastern China (Shandong, Henan, Hubei, Anhui, Zhejiang, and Jiangsu), and Southern China (Guangdong, Guangxi, Guizhou, Sichuan, Hunan, Chongqing, and Jiangxi). In the Northern China, PEDV, PoRV, and PDCoV had infection rates of 10.33% (224/2,169), 14.71% (319/2,169), and 11.66% (253/2,169), respectively (Figure 3B). Co-infection rates were 18.63% (404/2,169) for PEDV + PoRV, 0.60% (13/2,169) for PEDV + PDCoV, 10.28% (223/2,169) for PoRV + PDCoV, 0.55% (12/2,169) for PEDV + PDCoV + PoRV, respectively (Figure 3B). In the Mideastern China, PEDV, PoRV, and PDCoV had infection rates of 2.44% (40/1,642), 26.79% (440/1,642), and 3.59% (59/1,642), respectively (Figure 3B). Co-infection rates were 5.66% (93/1,642) for PEDV + PoRV, 0.06% (1/1,642) for PEDV + PDCoV, 2.68% (44/1,642) for PoRV + PDCoV, 1.22% (20/1,642) for PEDV + PDCoV + PoRV, respectively (Figure 3B). In the Southern China, PEDV, PoRV, and PDCoV had infection rates of were 5.16% (51/989), 28.51% (282/989), and 0.71% (7/989), respectively (Figure 3B). Co-infection rates were 7.18% (71/989) for PEDV + PoRV, 0 (0/989) for PEDV + PDCoV, 0.71% (7/989) for PoRV + PDCoV, 3.03% (30/989) for PEDV + PDCoV + PoRV, respectively (Figure 3B).

## 4 Discussion

Porcine viral diarrhea is a prevalent occurrence observed in clinical setting, resulting in substantial economic losses within the pig industry. Notably, PEDV, PoRV, and PDCoV are recognized as crucial pathogens causing this disease (Hou et al., 2023). With the development of large-scale and intensive swine production, mixed infections of these three pathogens are increasingly becoming common in swine farms (Huang et al., 2015). Detecting and identifying the pathogens quickly and accurately is crucial for preventing and controlling the spread of infectious diseases. Given the similarities in symptoms and pathology, as well as the frequent co-infection of these three viruses, it is challenging to differentiate them through clinical diagnosis alone (Vlasova et al., 2017; Shu et al., 2022). Hence, there is a need for a rapid and accurate method to simultaneously distinguish PEDV, PoRV, and PDCoV.

In this study, specific primers and probes were designed for the conserved regions of the PEDV M gene, PoRV NSP3 gene, and PDCoV M gene. After optimizing multiple times, a successful multiplex real-time PCR method was developed to simultaneously detect PEDV, PoRV, and PDCoV in a single amplification reaction. The method established in this study is highly sensitive, with a detection limit of 100 copies/μL. The multiplex real-time PCR also showed good repeatability, with coefficient of variation ranging from 0.11 to 0.87% for intra-assay and 0.11–1.63% for inter-assay. Thirty clinical samples with diarrhea symptoms were used to compare the results of the commercial singleplex real-time PCR kit and our multiplex real-time PCR method for detecting PEDV, PoRV, and PDCoV. The two methods showed complete agreement, indicating that the multiplex real-time PCR assay developed in this study could effectively replace the commercial singleplex real-time PCR kit for simultaneously differentiating PEDV, PoRV, and PDCoV.

Due to its rapid, highly sensitive, and specific characteristics, the multiplex real-time PCR assay established in this study has been widely used in early detection of pathogens in clinical samples. A total of 4,800 diarrhea samples from 18 provinces of china were used to investigate the prevalence of PEDV, PoRV, and PDCoV. Out of these samples, 2,207 were negative and 2,593 were positive for single or co-infections of these viruses. Among the positive samples, PoRV had the highest infection rate at 75% (1,945/2,593), followed by PEDV at 36.98% (959/2,593) and PDCoV at 25.8% (669/2,593), confirming PoRV as the primary cause of porcine diarrhea. These findings align with previous research showing a steady increase in PoRV infection rates, with some countries reporting 100% seropositivity in adult pigs (Pettersson et al., 2019).

Previous reports have shown that co-infection of these three viruses can lead to severe symptoms (Vlasova et al., 2017; Saeng-Chuto et al., 2021). Our study found that co-infections account for 35.4% (918/2,593) of positive samples, indicating that multiple pathogen co-infections are becoming more common with the development of large-scale and intensive swine production (Figure 3A). Specifically, co-infections of PoRV and PEDV accounted for 21.9% (568/2,593) of positive samples, followed by co-infections of PoRV and PDCoV at 10.57% (274/2,593; Figure 3A). Co-infections of PEDV and PDCoV were very rare, at only 0.54% (14/2,593; Figure 3A). Notably, 2.39% (62/2,593) of positive samples were co-infected with PEDV, PoRV, and PDCoV (Figure 3A). The findings align with previous studies indicating that the co-infection of PEDV and PoRV is the primary cause of porcine diarrhea in certain Chinese provinces (Zhang et al., 2019; Shu et al., 2022; Hou et al., 2023).

There were significant differences in positivity rate for these three viruses among the three regions: Northern China, Mideastern China), and Southern China. Northern China had the highest positivity rate for the three viruses at 66.76%, followed by Mideastern China at 42.40% and Southern China at 45.5% (Figure 3B). Northern China also had a higher incidence of co-infection at 45.03% compared to Mideastern China at 22.69% and Southern China at 24% (Figure 3B). However, Mideastern and Southern China had a higher positive rate of single PoRV than Northern China (Figure 3B). These results suggest that viral diarrhea disease in China pig farms is complex, possibly due to climate differences between the north and south and rapid pig population renewal in northern China. In summary, we have successfully developed a reliable multiplex real-time PCR assay for distinguishing PEDV, PoRV, and PDCoV. This assay has demonstrated excellent specificity, sensitivity, and repeatability. It has also been effectively used to detect clinical samples, making it a valuable tool for quickly identifying these viruses. Rapid and precise diagnostics, followed by prompt quarantine and treatment, could be helpful to prevent and control the spread of infectious diseases.

## Data availability statement

The original contributions presented in the study are included in the article/Supplementary material, further inquiries can be directed to the corresponding author.

## Author contributions

JR: Conceptualization, Funding acquisition, Writing—review & editing, Formal analysis, Methodology, Project administration, Writing—original draft. CZ: Writing—original draft, Data curation, Software. YL: Software, Writing—original draft, Resources. ML: Data curation, Writing—review & editing. JG: Data curation, Investigation, Writing—review & editing. FC: Data curation, Formal analysis, Writing—review & editing. XL: Conceptualization, Data curation, Funding acquisition, Resources, Writing—review & editing.
